# NT-proBNP levels and arrhythmias in adolescents with chronic upper gastrointestinal diseases

**DOI:** 10.1590/1984-0462/2026/44/2026053

**Published:** 2026-11-02

**Authors:** Natalia Shevchenko, Nataliia Krutenko, Tetiana Zimnytska, Kostiantyn Voloshyn, Tetiana Holovko

**Affiliations:** a V. N. Karazin Kharkiv National University, Kharkiv, Ukraine.

**Keywords:** Adolescent, Gastrointestinal diseases, Cardiac arrhythmias, Natriuretic peptide, brain, Electrocardiography, ambulatory, Adolescente, Gastroenteropatias, Arritmias cardíacas, Peptídeo natriurético encefálico, Eletrocardiografia ambulatorial

## Abstract

**Objective:**

To assess plasma N-terminal pro-B-type natriuretic peptide (NT-proBNP) levels in adolescents with chronic upper gastrointestinal diseases and investigate their association with 24-hour Holter electrocardiographic findings.

**Methods:**

This cross-sectional study included 104 adolescents with endoscopically-verified chronic upper gastrointestinal diseases and 58 comparison adolescents without clinically-documented chronic gastrointestinal or cardiovascular disease. Both groups underwent 24-hour Holter electrocardiographic monitoring. NT-proBNP was measured by enzyme-linked immunosorbent assay in 60 patients and 28 controls. Receiver operating characteristic analysis explored the ability of this biomarker to identify positive Holter findings, defined as rhythm, conduction, or repolarization abnormalities exceeding age-related physiological limits or requiring further cardiological evaluation.

**Results:**

Positive Holter findings were observed in 71 patients (68.3%). Most findings were benign; potentially clinically significant abnormalities occurred in 11 patients (10.6% of all patients; 15.5% of those with positive Holter findings). Median NT-proBNP levels did not significantly differ between patients and controls but were higher in patients with cardiovascular complaints (53.0 42.5-85.0 *versus* 22.6 12.4-41.4 pg/mL; p=0.001) and positive Holter findings (50.0 32.2-70.5 *versus* 27.8 10.7-41.4 pg/mL; p<0.05). According to the receiver operating characteristic analysis for identifying positive Holter findings, we observed an area under the curve of 0.779 (95% confidence interval 95%CI: 0.726-0.833; p<0.001). An NT-proBNP cutoff value of ≥43.2 pg/mL showed 66.7% sensitivity and 90.9% specificity for identifying positive Holter findings.

**Conclusions:**

Higher NT-proBNP levels were associated with positive Holter findings. Given the cross-sectional design and limited monitoring period, this association requires prospective validation.

## RESUMO

**Objetivo:**

Avaliar os níveis plasmáticos do fragmento N-terminal do propeptídeo natriurético tipo B (NT-proBNP) em adolescentes com doenças crônicas do trato gastrointestinal superior e sua associação com achados da monitorização Holter de 24 horas.

**Métodos:**

Estudo transversal com 104 adolescentes com doenças crônicas do trato gastrointestinal superior confirmadas por endoscopia e 58 adolescentes sem doença gastrointestinal ou cardiovascular crônica documentada. Todos foram submetidos à monitorização Holter de 24 horas. O NT-proBNP foi dosado por ensaio imunoenzimático em 60 pacientes e 28 controles. A análise da curva característica de operação do receptor (ROC) explorou a capacidade desse biomarcador de identificar achados positivos no Holter, definidos como alterações de ritmo, condução ou repolarização que excediam os limites fisiológicos relacionados à idade ou exigiam avaliação cardiológica adicional.

**Resultados:**

Achados positivos no Holter foram observados em 71 pacientes (68,3%). A maioria foi benigna; alterações potencialmente clinicamente significativas foram observadas em 11 pacientes (10,6%). Os níveis medianos de NT-proBNP não diferiram entre pacientes e controles, mas foram maiores em pacientes com queixas cardiovasculares (53,0 [42,5-85,0 ] *versus* 22,6 [12,4-41,4 ] pg/mL; p=0,001) e naqueles com achados positivos no Holter (50,0 [32,2-70,5 ] *versus* 27,8 [10,7-41,4 ] pg/mL; p<0,05). A área sob a curva ROC foi 0,779 (intervalo de confiança de 95% [IC95% ]: 0,726-0,833; p<0,001). O ponto de corte de ≥43,2 pg/mL apresentou sensibilidade de 66,7% e especificidade de 90,9%.

**Conclusões:**

Níveis mais elevados de NT-proBNP estiveram associados a achados positivos no Holter. Considerando o delineamento transversal e o período limitado de monitorização, essa associação requer validação prospectiva.

## INTRODUCTION

Gastrointestinal (GI) diseases remain one of the leading challenges of modern medicine, characterized by high global prevalence and substantial socioeconomic consequences.^1^ According to a systematic analysis of the *Global Burden of Diseases* study covering 204 countries, the prevalence of digestive system disorders has not shown a significant decline over nearly 30 years of observation.^2^ The high incidence of these conditions among the pediatric population is particularly worrisome, especially during adolescence.^3^ ^,^ ^4^ ^,^ ^5^ Comorbidity of gastrointestinal diseases with cardiovascular disorders, as well as metabolic and psychoneurological conditions, is regarded as one of the key medical and social problems in contemporary pediatrics.^1^ ^,^ ^6^ ^,^ ^7^ ^,^ ^8^ ^,^ ^9^ ^,^ ^10^ Jie Chen et al. (2024), in a large-scale prospective cohort study, confirmed a statistically significant association between cardiovascular pathology and chronic gastrointestinal diseases, including gastritis and duodenitis, gastroesophageal reflux disease, peptic ulcer disease, and Barrett’s esophagus.^11^ During adolescence, the interplay between the gastrointestinal tract and the cardiovascular system is of particular clinical relevance, as functional and inflammatory gastrointestinal processes may be accompanied by autonomic dysfunction, altered cardiac rhythm regulation, and the development of arrhythmias.^12^ ^,^ ^13^ ^,^ ^14^ ^,^ ^15^ ^,^ ^16^

Among cardiac biomarkers, N-terminal pro-B-type natriuretic peptide (NT-proBNP) is widely used to assess myocardial wall stress and has diagnostic and prognostic value in cardiovascular conditions. Although NT-proBNP is primarily recognized as a biomarker of heart failure, authors of contemporary studies suggest that it may also reflect subclinical myocardial stress and cardiovascular risk in selected populations.^17^ ^,^ ^18^ ^,^ ^19^ ^,^ ^20^ ^,^ ^21^ ^,^ ^22^ ^,^ ^23^ Muresan et al. reported higher NT-proBNP levels in patients with ventricular arrhythmias than in those without rhythm disturbances, suggesting a possible association between this biomarker and clinically relevant arrhythmic findings.^24^ Similar pediatric findings were reported by Mazurek et al., who found higher NT-proBNP levels in children with potentially clinically significant ventricular arrhythmias than in those with mild arrhythmias.^25^

Therefore, assessing NT-proBNP in adolescents with chronic gastrointestinal diseases may contribute to the exploratory evaluation of cardiovascular findings, but its diagnostic or prognostic role requires cautious interpretation and further validation.

In the present study, we aimed to assess plasma NT-proBNP levels in adolescents with chronic upper GI diseases and to investigate their association with rhythm, conduction, or repolarization abnormalities detected during 24-hour Holter electrocardiographic (ECG) monitoring.

## METHOD

The research was conducted at a tertiary pediatric healthcare center and a municipal children’s hospital during 2020-2024. The study had a cross-sectional design with two components:

1. A prevalence study to identify Holter ECG abnormalities among adolescents with chronic upper gastrointestinal diseases within the target clinical population, and
2. A comparative cross-sectional analysis between adolescents with chronic upper gastrointestinal diseases and a disease-free comparison group.

The study was reported in accordance with the Strengthening the Reporting of Observational Studies in Epidemiology (STROBE) statement for cross-sectional studies.

The study included 162 adolescents aged 10-18 years: 104 patients with chronic inflammatory upper GI diseases, including chronic gastroduodenitis (CGD), gastroesophageal reflux disease (GERD), and gastric or duodenal ulcer disease, and 58 comparison adolescents without clinically-documented chronic gastrointestinal or cardiovascular disease.

No formal a priori sample size calculation was performed before recruitment. Sample size adequacy for the prevalence component was assessed *post hoc* using the single-proportion formula n=Z^2^×p×(1−p)/d^2^. With a 95% confidence level, a conservative expected proportion of 50%, and an absolute precision of 10 percentage points, the minimum required sample size was 97 adolescents; therefore, the final study group of 104 adolescents with chronic upper GI diseases was considered adequate for estimating the prevalence of Holter ECG abnormalities. Biomarker, receiver operating characteristic (ROC), and subgroup analyses were considered exploratory.

All adolescents examined at the participating centers during the study period were considered for inclusion if they met the eligibility criteria and had complete core clinical and Holter ECG data. No additional sampling procedure was applied. No eligible adolescent refused participation or was excluded because of withdrawal of consent, incomplete Holter ECG recordings, or missing key clinical data.

The protocol was approved by the local institutional bioethics committee (Protocol No. 5/1, February 19, 2025) and complied with the principles of the Declaration of Helsinki. Written Informed Consent was obtained from all participants and/or their legal representatives before enrollment.

General eligibility criteria for both groups were age 10-18 years, moderate physical activity without regular sports training, and absence of previously documented clinically significant rhythm, conduction, or repolarization abnormalities. Exclusion criteria for both groups were congenital or acquired structural heart defects, acute infectious conditions, endocrine or respiratory diseases, use of medications affecting cardiac rhythm, and chronic heart failure.

The study-group-specific criterion was endoscopically-verified chronic inflammatory upper GI disease. The comparison group was recruited from adolescents who underwent routine clinical evaluation at the participating centers and had no clinically-documented chronic gastrointestinal or cardiovascular disease according to medical history, available medical records, and clinical evaluation.

For the GI assessment, patients in the study group underwent clinical and laboratory evaluation, video esophagogastroduodenoscopy (VEGDS) using an Olympus CV-140 endoscope, intragastric pH-metry, and assessment of *Helicobacter pylori* infection. GERD and reflux esophagitis were classified according to the Savary-Miller classification modified by Tytgat. Gastroduodenal mucosal changes were classified according to the Sydney-Houston classification and the modified Sydney classification for pediatric gastropathy. Endoscopic findings were additionally categorized as erythematous/nondestructive changes without visible erosions or ulceration, or erosive-ulcerative changes defined by the presence of erosions and/or gastric or duodenal ulcerative lesions. This subdivision was exploratory and was used to assess whether mucosal lesion type was associated with NT-proBNP levels or Holter-detected abnormalities. VEGDS was not systematically performed in the control group because there were no clinical indications for endoscopic examination.

Both patients and controls underwent 24-hour Holter electrocardiographic monitoring (Holter ECG) using a CONTEC TLC5000 device and ECG Holter System_TF(OS) V5.5.2.5 software. Holter ECG recordings were analyzed according to international recommendations for ambulatory ECG monitoring and pediatric arrhythmia assessment.^26^ ^,^ ^27^ For analysis purposes, Holter ECG findings were classified as negative when all detected phenomena were within age-related physiological limits and were not considered clinically significant. Findings that exceeded age-related physiological limits or required further cardiological evaluation were classified as positive. Holter ECG recordings were interpreted by specialists who were independent of the laboratory measurement of NT-proBNP concentrations; however, formal blinding to NT-proBNP levels and clinical grouping was not applied.

Plasma NT-proBNP levels were measured in a subset of 88 adolescents, including 60 patients with chronic upper GI diseases and 28 comparison adolescents, using venous blood samples and a sandwich Enzyme-Linked Immunosorbent Assay (ELISA) with a commercial FineTest kit (China; catalog No. EH0350), according to the manufacturer’s protocol. Blood samples were collected in the morning after an overnight fasting.

Statistical analysis was performed using Excel for Windows, OpenOffice software, and the licensed statistical packages STATISTICA 6.1 and STATA/SE 12.0 for Windows. Categorical variables were presented as absolute numbers and percentages (n, %) and compared using Fisher’s exact test. Continuous variables were expressed as mean ± standard deviation or median (Me) and interquartile range (Q25-Q75). ROC analysis was used to assess the ability of NT-proBNP to identify Holter ECG abnormalities; the area under the curve, sensitivity, specificity, and the optimal cutoff value based on the Youden index were calculated. A p-value <0.05 was considered statistically significant.

## RESULTS

The study included 104 adolescents aged 10-18 years (mean age, 15.11±1.99 years) diagnosed with chronic upper GI diseases. The distribution of gastrointestinal diseases by sex is presented in Table 1.

**Table 1.** Distribution of gastrointestinal diseases according to sex.

Parameter	Boys (n=62; %)	Girls (n=42; %)	Total (n=104; %)
CGD	38 (61.3)*	34 (81.0)	72 (69.3)
GERD	18 (29.0)	8 (19.1)	26 (25.0)
GU/DU	6 (9.7)	0 (0.0)	6 (5.8)

Note: Data are presented as number (n) and percentage (%).

*p<0.05.

CGD: Chronic gastroduodenitis; GERD: Gastroesophageal reflux disease; GU: Gastric ulcer; DU: Duodenal ulcer.

Source: Authors’ own data.

According to VEGDS findings, erosive-ulcerative lesions were more common in adolescents with GERD than in those with CGD (50.0%; p<0.05).

According to intragastric pH-metry data, increased gastric acid-secreting function was detected in 61 adolescents (58.7%), whereas preserved gastric acid secretion was observed in 43 adolescents (41.3%). Increased gastric acid-producing function was more frequent in adolescents with GERD than in those with CGD (73.1% vs. 43.8%; p=0.01).

During 24-hour Holter ECG monitoring, a variety of electrophysiological phenomena were identified in adolescents from the study group. In 33 patients (31.7%), Holter ECG findings were classified as negative because the detected phenomena were within age-related physiological limits. This category included sinus arrhythmia, wandering atrial pacemaker, ectopic atrial rhythm with an appropriate heart rate, ≤50 supraventricular or isolated ventricular premature beats per day, transient sinoatrial block, first-degree atrioventricular block, borderline QTc interval, and occasional nocturnal rhythm pauses.

Holter ECG recordings were classified as positive in 71 patients (68.3%) when rhythm, conduction, or repolarization findings exceeded age-related physiological limits or were considered potentially clinically significant. These included inappropriate sinus tachycardia or bradycardia, frequent supraventricular or ventricular premature beats, accelerated ectopic rhythms, ventricular tachycardia runs, second-degree sinoatrial or atrioventricular block, prolonged QTc interval, age-inappropriate rhythm pauses, T-wave inversion in standard limb leads, and early ventricular repolarization pattern.

Holter-detected abnormalities were more frequent in adolescents with nondestructive mucosal changes than in those with erosive-ulcerative lesions (p<0.05; Table 2).

**Table 2.** Frequency of Holter electrocardiographic abnormalities according to endoscopic findings.

Parameter	Nondestructive changes (n=62; %)	Erosive-ulcerative changes (n=42; %)
Positive Holter ECG test	48 (77.4%)*	23 (54.8%)
Negative Holter ECG test	14 (22.6%)	19 (45.2%)

Note: Data are presented as number (n) and percentage (%).

*p<0.05.

ECG: Electrocardiography.

Source: Authors’ own data.

At the same time, no statistically significant differences were observed depending on the clinical variant of gastrointestinal disease (p>0.05). Holter-detected abnormalities were found in 52 of 72 adolescents with CGD (72.2%), 16 of 26 adolescents with GERD (61.5%), and three of six adolescents with gastric or duodenal ulcer (50.0%).

In Table 3, we present the distribution of Holter-positive patients according to benign and potentially clinically significant findings. The latter included accelerated ectopic rhythms, ventricular tachycardia runs, second-degree sinoatrial or atrioventricular block, and prolonged QTc interval. These findings were recorded in 11 adolescents, representing 10.6% of the total study group (Table 3).

**Table 3.** Frequency of benign and potentially clinically significant Holter-detected findings by clinical variant of gastrointestinal tract disease.

Type of arrhythmia	Clinical variant of GIT involvement			Total (positive Holter test) (n=71; %)
	CGD (n=52; %)	GERD (n=16; %)	GU/DU (n=3; %)	
Benign arrhythmias	45 (86.5)	13 (81.3)	2 (66.7)	60 (84.5)
Potentially clinically significant arrhythmias	7 (13.5)	3 (18.8)	1 (33.3)	11 (15.5)

Note: Data are presented as number (n) and percentage (%).

GIT: Gastrointestinal tract; CGD: Chronic gastroduodenitis; GERD: Gastroesophageal reflux disease; GU: Gastric ulcer; DU: Duodenal ulcer.

Source: Authors’ own data.

In the control group, Holter ECG phenomena were detected in 29 of 58 adolescents (50.0%), which was significantly less frequent than in the study group (68.3%; p=0.028); all findings in controls were interpreted as benign or age-related physiological variants, with no potentially clinically significant arrhythmias recorded. Detailed comparative Holter ECG data have been previously reported.^15^

In the NT-proBNP subset, median levels did not significantly differ between patients and comparison adolescents (33.3 16.2-54.8 vs. 27.1 12.7-45.0 pg/mL; p>0.05). No significant differences in NT-proBNP levels were observed across the demographic and clinical characteristics presented in Table 4 (all p>0.05). However, patients with erosive-ulcerative lesions had higher NT-proBNP levels than comparison adolescents (44.7 [12.9-78.5] vs. 27.1 [12.7-45.0] pg/mL; p<0.05).

**Table 4.** Plasma N-terminal pro-B-type natriuretic peptide levels in patients with chronic upper gastrointestinal diseases (n=60) according to demographic and clinical characteristics.

Parameter	n	NT-proBNP (pg/mL), median [Q25-Q75]
Sex		
Boys	39	35.1 16.8-54.8
Girls	21	41.3 23.3-62.0
Adolescence period		
Early adolescence	9	59.7 24.5-242.9
Middle adolescence	26	40.3 18.4-53.0
Late adolescence	25	29.8 15.8-53.8
Disease duration (years)		
≤1	27	40.3 14.5-53.8
1-3	18	41.3 15.8-75.3
≥3	15	26.3 18.4-43.6
Endoscopic variant		
Erosive-ulcerative changes	23	44.7 12.9-78.5
Nondestructive changes	37	32.2 18.9-53.0
Clinical variant		
Chronic gastroduodenitis	42	27.8 16.5-62.0
Gastroesophageal reflux disease	14	41.9 18.5-51.2
Gastric ulcer/duodenal ulcer	4	32.9 15.8-50.0

Note: Data are presented as median [Q25-Q75]. NT-proBNP: N-terminal pro-B-type natriuretic peptide.

Source: Authors’ own data.

Cardiovascular complaints were reported by 30 of 104 adolescents (28.8%). Patients with cardiovascular complaints had higher NT-proBNP concentrations than those without complaints (53.0 42.5-85.0 vs. 22.6 12.4-41.4 pg/mL; p=0.001). NT-proBNP concentrations were also higher in patients with positive Holter findings than in those with negative Holter findings (50.0 [32.2-70.5] vs. 27.8 [10.7-41.4] pg/mL; p=0.0489; Figure 1).

**Figure 1.**
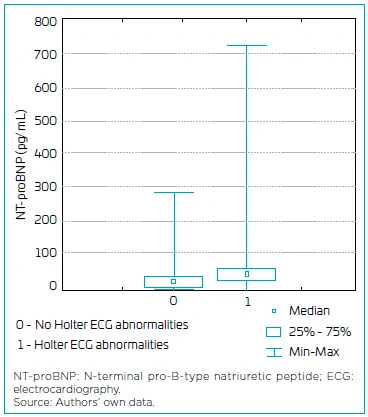
Plasma N-terminal pro-B-type natriuretic peptide levels according to the presence of Holter electrocardiographic abnormalities. Data are presented as median and interquartile range (25th-75th percentiles) with minimum and maximum values. p=0.0489 (Mann-Whitney test).

ROC analysis was used as an exploratory assessment of the association between NT-proBNP levels and positive Holter findings in this cohort. The area under the ROC curve was 0.779 (standard error [SE]=0.027; 95% confidence interval [95%CI\: 0.726-0.833; p<0.001). The optimal NT-proBNP cutoff value of ≥43.2 pg/mL showed 66.7% sensitivity and 90.9% specificity, with a corresponding Youden index of 0.576 (Figure 2).

**Figure 2.**
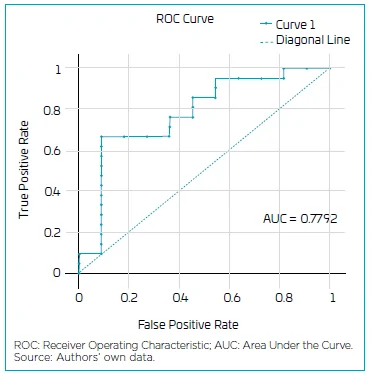
Receiver operating characteristic curve for NT-proBNP in identifying positive Holter findings. The diagonal line represents no discrimination (AUC=0.5).

## DISCUSSION

In the present study, we found an association between plasma NT-proBNP levels and rhythm, conduction, or repolarization abnormalities detected during 24-hour Holter ECG monitoring in adolescents with chronic upper GI diseases. However, due to the cross-sectional design, the present data demonstrate association rather than prediction and do not establish temporal or causal relationships between gastrointestinal disease, NT-proBNP concentrations, and Holter ECG findings.

The clinical relevance of the detected Holter ECG findings should be cautiously interpreted. In adolescents, isolated premature beats, mild conduction abnormalities, transient rhythm changes, and some repolarization patterns may be benign, particularly in the absence of structural heart disease, hemodynamic instability, or clinically significant symptoms. In the present cohort, no life-threatening arrhythmias or hemodynamic disturbances were reported. Therefore, the findings should not be interpreted as evidence of severe cardiac involvement, but rather as functional or potentially clinically relevant cardiovascular findings requiring individualized interpretation.

The limited duration of Holter ECG monitoring is also important for interpretation. A normal 24-hour recording cannot exclude intermittent arrhythmias occurring outside the monitoring period, whereas abnormalities detected during a single recording may reflect transient autonomic or physiological influences. Therefore, patients with negative Holter ECG recordings were described as having no clinically relevant abnormalities during the monitoring period, rather than as having no arrhythmias.

Because no clinically significant rhythm, conduction, or repolarization abnormalities had been documented before enrollment, the Holter ECG findings observed in this cohort may reflect functional cardiovascular disturbances associated with chronic upper GI diseases. Possible mechanisms include autonomic imbalance, neurohumoral activation, and systemic inflammation.^16^ ^,^ ^19^ ^,^ ^28^ Nevertheless, previously unrecognized individual ECG features cannot be excluded; therefore, prolonged QTc interval, T-wave inversion, and early ventricular repolarization pattern should be interpreted as associated cardiovascular findings rather than proven consequences of gastrointestinal inflammation.

Comparable observations have been reported in pediatric populations, in which many rhythm findings are considered benign or age-related physiological variants.^29^ Mikhieieva and Nechytailo reported cardiac arrhythmias in 40.0% of children with chronic gastrointestinal disorders and conduction abnormalities in 15.6%.^14^ Data from the Multi-Ethnic Study of Atherosclerosis (MESA) cohort suggest that elevated NT-proBNP may reflect subclinical myocardial stress, including asymptomatic left ventricular dysfunction and myocardial fibrosis; however, these adult data cannot be directly extrapolated to adolescents.^17^ ^,^ ^21^ Similar pediatric findings were reported by Mazurek et al., who found higher NT-proBNP levels in children with potentially clinically significant ventricular arrhythmias than in those with mild arrhythmias.^25^

In the present study, the exploratory NT-proBNP threshold of ≥43.2 pg/mL was derived from this specific clinical cohort and should not be extrapolated to other adolescent populations without external validation. Therefore, NT-proBNP should be regarded as a potential auxiliary marker rather than a validated tool for arrhythmia risk stratification.

An apparently paradoxical observation was the higher frequency of Holter-detected abnormalities in adolescents with erythematous/nondestructive changes than in those with erosive-ulcerative lesions. This result should be interpreted with caution because the ulcer subgroup was small and the estimate may be unstable. In addition, 24-hour Holter monitoring reflects rhythm findings only during a limited recording period, and the timing of monitoring in relation to gastrointestinal disease activity may have influenced the results.

This study has several limitations. First, its cross-sectional design does not allow assessment of temporality or causality. Second, the limited regional clinical setting may restrict generalizability. Third, although the sample size was considered adequate for the prevalence component, subgroup, ROC, and biomarker-related analyses were exploratory because NT-proBNP was measured in 60 patients and 28 controls rather than in the entire study population. Fourth, formal blinding of Holter ECG interpretation to NT-proBNP levels and clinical grouping was not applied, which may have introduced observer bias. Fifth, VEGDS was not systematically performed in comparison adolescents because there were no clinical indications; therefore, subclinical GI lesions cannot be definitively excluded. Finally, 24-hour Holter ECG monitoring cannot exclude intermittent arrhythmias outside the recording period, and the NT-proBNP threshold derived from ROC analysis was not externally validated.

In conclusion, Holter-detected rhythm, conduction, or repolarization abnormalities were frequent among adolescents with chronic upper GI diseases. Although NT-proBNP concentrations were comparable between patients and comparison adolescents, higher NT-proBNP levels within the patients’ group were associated with Holter-detected abnormalities. Because of the cross-sectional design, these findings indicate association only and require prospective validation.

## Data Availability

The database that originated the article is available with the corresponding author.
